# Simultaneous Measurement of Kidney Function by Dynamic Contrast Enhanced MRI and FITC-Sinistrin Clearance in Rats at 3 Tesla: Initial Results

**DOI:** 10.1371/journal.pone.0079992

**Published:** 2013-11-18

**Authors:** Frank G. Zöllner, Daniel Schock-Kusch, Sandra Bäcker, Sabine Neudecker, Norbert Gretz, Lothar R. Schad

**Affiliations:** 1 Computer Assisted Clinical Medicine, Medical Faculty Mannheim, Heidelberg University, Mannheim, Germany; 2 Medical Research Center, Medical Faculty Mannheim, Heidelberg University, Mannheim, Germany; National Institute of Radiological Sciences, Japan

## Abstract

Glomerular filtration rate (GFR) is an essential parameter of kidney function which can be measured by dynamic contrast enhanced magnetic resonance imaging (MRI-GFR) and transcutaneous approaches based on fluorescent tracer molecules (optical-GFR). In an initial study comparing both techniques in separate measurements on the same animal, the correlation of the obtained GFR was poor. The goal of this study was to investigate if a simultaneous measurement was feasible and if thereby, the discrepancies in MRI-GFR and optical-GFR could be reduced. For the experiments healthy and unilateral nephrectomised (UNX) Sprague Dawley (SD) rats were used. The miniaturized fluorescent sensor was fixed on the depilated back of an anesthetized rat. A bolus of 5 mg/100 g b.w. of FITC-sinistrin was intravenously injected. For dynamic contrast enhanced perfusion imaging (DCE-MRI) a 3D time-resolved angiography with stochastic trajectories (TWIST) sequence was used. By means of a one compartment model the excretion half-life (t1/2) of FITC-sinistrin was calculated and converted into GFR. GFR from DCE-MRI was calculated by fitting pixel-wise a two compartment renal filtration model. Mean cortical GFR and GFR by FITC-sinistrin were compared by Bland-Altman plots and pair-wise t-test. Results show that a simultaneous GFR measurement using both techniques is feasible. Mean optical-GFR was 4.34±2.22 ml/min (healthy SD rats) and 2.34±0.90 ml/min (UNX rats) whereas MRI-GFR was 2.10±0.64 ml/min (SD rats) and 1.17±0.38 ml/min (UNX rats). Differences between healthy and UNX rats were significant (p<0.05) and almost equal percentage difference (46.1% and 44.3%) in mean GFR were assessed with both techniques. Overall mean optical-GFR values were approximately twice as high compared to MRI-GFR values. However, compared to a previous study, our results showed a higher agreement. In conclusion, the possibility to use the transcutaneous method in MRI may have a huge impact in improving and validating MRI methods for GFR assessment in animal models.

## Introduction

Renal diseases can lead to terminal kidney failure that requires life-long dialysis or renal transplantation. Early detection and treatment can delay or even prevent the progression towards end stage renal disease (ESRD). However, most renal and renovascular diseases like renal artery stenosis or renal parenchymal disease do not initially produce symptoms or pain. Therefore, it is important to monitor renal function closely in populations at high risk to develop kidney diseases [1].

Renal perfusion and glomerular filtration rate (GFR) are essential parameters of kidney function. Several tracers are available for GFR evaluation, including endogenous markers such as creatinine and cystatin C, or exogenous markers, including iothalamate [2] or inulin, which is accepted as gold standard for determination of renal function. Also available are radioactive labeled tracers like C^51^-ethylenediaminetetraacetic acid (EDTA) or Tc^99m^-diethylene triamine pentaacetic acid (DTPA) [3], [4]. Current techniques for the measurement of GFR, such as clearance of inulin or sinistrin, scintigraphy with radio-labeled markers, and creatinine clearance are limited. Either they are invasive, expensive, result in a radiation exposure, or are inaccurate, e.g. because of serum creatinine dependency on muscular tissue mass and nutritional factors [5], [6]. Thus, at the present time, GFR is only approximated using approaches such as the Chronic Kidney Disease Epidemiology Collaboration (CKD-EPI) or Modification of Diet in Renal Disease (MDRD) [7], [8].

Recent approaches attempting to overcome some of the mentioned limitations of GFR measurement techniques are dynamic contrast enhanced magnetic resonance imaging (DCE-MRI) [9], [10], [11] or transcutaneous approaches based on fluorescent tracer molecules [12], [13], [14]. Both approaches allow for a non-invasive and radiation free way to measure GFR. Furthermore, DCE-MRI also allows for single kidney GFR and even spatially resolved analysis of the GFR through calculation of parametric maps.

Although these techniques are promising there are limitations. Quantitative DCE-MRI is possible but is rarely used in clinical settings since validation is difficult [15]. In those studies in which a direct comparison with gold standard measurements was performed, only a poor correlation was found in human as well as in animal studies [15].

In contrast to DCE-MRI, none of the transcutaneous techniques have been used in humans as the tracers are not yet approved for human use. However, the fluorescence based transcutaneous techniques were directly validated against classical bolus clearance techniques in animal models [12], [13], [14]. Combining both techniques, however, allows for validation of DCE-MRI by fluorescence based transcutaneous optical imaging in animal models. In an initial study, Sadick et al. compared a transcutaneous and a DCE-MRI technique in rats [16]. The correlation of the obtained GFR measures within healthy rats and a rat model of polycystic kidney disease was poor. However, the study had some limitations: the optical and MRI measurements were performed on different days, as the optical method could not be performed in the MRI at that time, introducing probably a systematic error as the animals had to be anaesthetized twice.

In this study a miniaturized sensor for the transcutaneous measurement of FITC-sinistrin elimination kinetics, recently introduced for measurements in conscious mice [17], was improved to enable simultaneous MRI and fluorescent imaging. Furthermore, the DCE-MRI protocol was modified to allow for 3D time resolved imaging with high temporal and spatial resolution in order to improve measurement results in rats. This setup allowed, for the first time, a simultaneous measurement of GFR by DCE-MRI and transcutaneous optical imaging. Both methods are independent of blood and urine sampling as well as from laboratory assays, making them convenient for the experimenter. The goal was to investigate if a simultaneous measurement was feasible and if thereby, the discrepancies in MRI-GFR and optical-GFR could be reduced.

## Materials and Methods

### Animals

The study was conducted in accordance with federal and local laws. For the experiments healthy (n = 6; 382±62 g b.w.) and unilateral nephrectomised (UNX) (n = 6; 383±73 g b.w.) Sprague Dawley (SD) rats were used. Rats were purchased from Janvier, France. All animals had free access to standard food and water (Ssniff GmbH experimental animal diet R/M-H).

### Ethics

All procedures were performed in accordance with the Guide of the Care and Use of Laboratory Animals published by the National Academy of Sciences and were approved by the local authorities (Regional council Karlsruhe, G235/10).

### Animal preparation

For FITC-sinistrin and Dotarem injection, a catheter was inserted into the femoral vein under subcutaneous MMF anesthesia (Midazolam 2 mg/kg body weight (b.w.)/Medetomidine 0.15 mg/kg b.w./Fentanyl 0.005 mg/kg b.w.) and exteriorized at the back of the neck. Unilateral nephrectomy (UNX) of the left kidney was performed as published before [18]. Catheter and UNX surgery were performed the day before the GFR measurement.

### MRI

All measurements were performed on a 3T whole-body MR scanner (Magnetom Tim Trio, Siemens Healthcare Sector, Erlangen, Germany) operating at a maximum gradient strength of 45 mT/m and a maximum slew rate of 200 T/m/s. An eight channel receive-only volumetric rat array (RAPID Biomedical GmbH, Rimpar, Germany) was used for signal detection. The body coil was used for homogeneous radio frequency (RF) transmission.

### MR compatibility of the optical sensor

Prior studies have not evaluated the simultaneous use of the optical device for FITC-sinistrin clearance and the MRI. Given that radiofrequency (RF) pulses, gradients or the magnetic field strength could cause interferences, we checked the device within the scanner for proper operation We also investigated whether the optical device created MR image artifacts [19]. The optical sensor was attached to a falcon tube (filled with water) and placed inside the receive coil and the magnet. The optical device was switched on in order to record signals while several image acquisitions were run. After the measurements, the acquired images were inspected visually for image artefacts and the recorded data was transferred to an offline workstation for analysis of the recorded signal.

### Simultaneous MRI and FITC-sinistrin clearance measurement

The transcutaneous FITC-sinistrin clearance was performed as previously described [17], [20].

The miniaturized fluorescent sensor (NIC-Kidney, Mannheim Pharma & Diagnostics GmbH, Mannheim, Germany) was fixed on the depilated back of an anesthetized rat using a double-sided adhesive patch (Lohmann GmbH & Co. KG, Neuwied, Germany); the fluorescence background level of the skin is recorded for one minute. Thereafter, a bolus of 5 mg/100 g b.w. of FITC-sinistrin dissolved in 0.9% NaCl solution (Mannheim Pharma & Diagnostics GmbH, Mannheim Germany) was intravenously injected. The catheter was flushed with 0.6 ml 0.9% NaCl solution (Delta Select GmbH, Rimbach, Germany) to assure complete FITC-sinistrin injection. The measurement time was 90 min with measurement intervals of one second.

Immediately after injecting the fluorescence tracer, the MRI acquisition was started. After localization, high resolution morphological images were acquired using a 2D turbo spin echo (TSE) sequence. Imaging parameters were as follows: repetition time (TR)  =  6980 ms, echo time (TE)  =  80 ms, echo train length (ETL)  =  28, flip angle of 140°, field of view 65×87 mm^2^, number of averages  =  12. The matrix size was 320×240×20 resulting in a voxel resolution of 0.3×0.3m×1.2 mm.

For DCE-MRI a 3D time-resolved angiography with stochastic trajectories (TWIST) sequence [21] optimized for renal perfusion in small animals was utilized [22]. The following sequence parameters were used: TR  =  3.4 ms, TE  =  1.4 ms, a flip angle of 20°, field of view 114×50 mm^2^, and generalized autocalibrating partially parallel acquisition (GRAPPA) of factor 2. Matrix size was 192×84×28 and voxel resolution was 0.6×0.6×1.2 mm^3^. TWIST view sharing was set to 15% central region and 20% sampling density in the outer region. The nominal temporal resolution was 0.9 sec per volume. Images were continuously acquired for 7 minutes. According to a normal clinical dose and the manufacturer’s instruction about 0.05 ml of contrast agent (Dotarem®, Guerbet, Roissy CdG Cedex, France), followed by a saline flush of 0.6 ml, was administered manually after the 15th volume was acquired. All MR imaging was performed in transversal slice orientation and slice positioning was copied from the morphological scan.

### Data processing

**FITC-sinistrin clearance.** By means of a one compartment model the excretion half-life (t_1/2_) of FITC-sinistrin was calculated and converted into GFR by a previously described conversion equation [13]



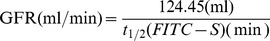
. 

**MRI.** Quantification of DCE-MRI kidney filtration was performed by fitting a published two compartment renal filtration model [23] using an in-house OsiriX plug-in [24]. The software calculates maps of the tubular flow (ml/100 ml/min) i.e. the flow of the tracer from the vascular to the tubular compartment corresponding to the filtration of the contrast agent from the blood stream. The arterial input function (AIF) was determined by carefully placing a ROI in the abdominal aorta distal to the branch of the renal arteries to avoid inflow effects and to minimize partial volume effects due to the small vessel diameter. All data were normalised by subtracting the mean intensity of 15 baseline volumes, and a linear relationship of the contrast agent concentration to the measured signal intensities was assumed [25]. To support this assumption, a phantom consisting of tubes filled with water and different concentrations of Dotarem (0 – 2.5 mmol/ml) was imaged using the above described DCE-MRI sequence. ROIs with size of 0.3 mm^2^ were placed inside each tube to measure the signal intensity and a plot of concentration versus signal intensity was plotted.

Total GFR for each kidney was estimated by using the equation of GFR  =  tubular flow * cortex volume as described by [16], [26]. Therefore, the T2 weighted images were used to measure the volume of the renal cortex. Cortex segmentation was performed by manual delineations for each renal cortex and slice. To estimate the tubular flow for each kidney, the calculated maps and the T2 weighted images were co-aligned and the derived cortical ROIs were copied into the tubular flow maps. To estimate whole kidney GFR, the obtained values for each kidney were added.

### Statistical Analysis

To compare the two methods and to evaluate whether systematic errors exist, calculated GFR values were plotted using a Bland-Altman plot. To further investigate the differences in GFR in healthy and UNX rats within each measurement technique, a paired t-test was employed. A difference in GFR was considered significant at p<0.05.

## Results

**Figure 1:**
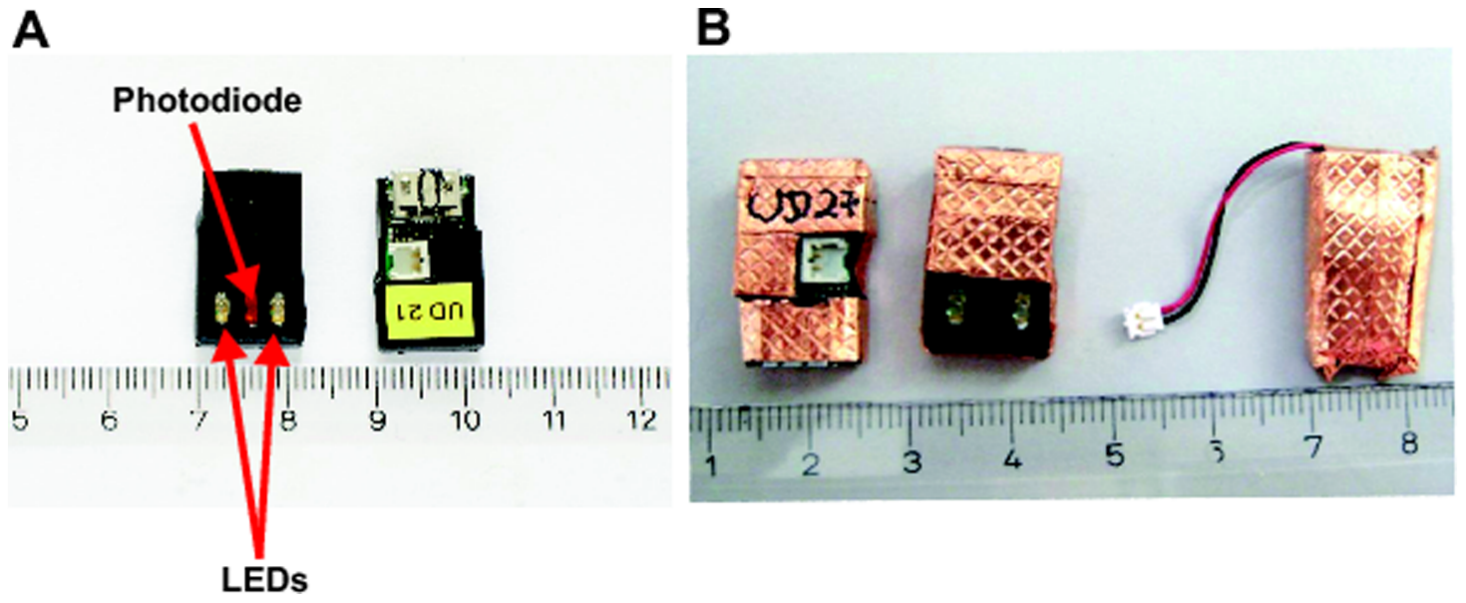
Optical devices for FITC-sinistrin clearance measurement. A: original devices with LEDs and photodiode for signal excitation and reception (bottom side) and electronics (upper side). B: the devices are shielded by copper foil, only the LEDs, the photodiode and the battery plug are spared. The device is powered by a small battery that was covered in copper foil, too.

At first, phantom experiments were performed to test MR compatibility of the optical sensor. The phantom experiment showed that the optical device causes signal cancelation. Therefore, we placed the optical device on the animal as far away as possible from the kidneys. Regarding the functioning of the optical device during image acquisition we observed that the RF or gradient pulses interact with the electric circuits of the optical sensor resetting the device a few minutes after starting the MR measurement, thereby causing a complete loss of recorded data. To overcome this problem the device was shielded by a thin copper foil exposing only the LEDs, the photodiode, and the battery plug (Fig. 1). The shielding prevented data loss of the optical device thereafter. The field strength of 3 Tesla had no effect on the functionality of the optical device.

**Figure 2:**
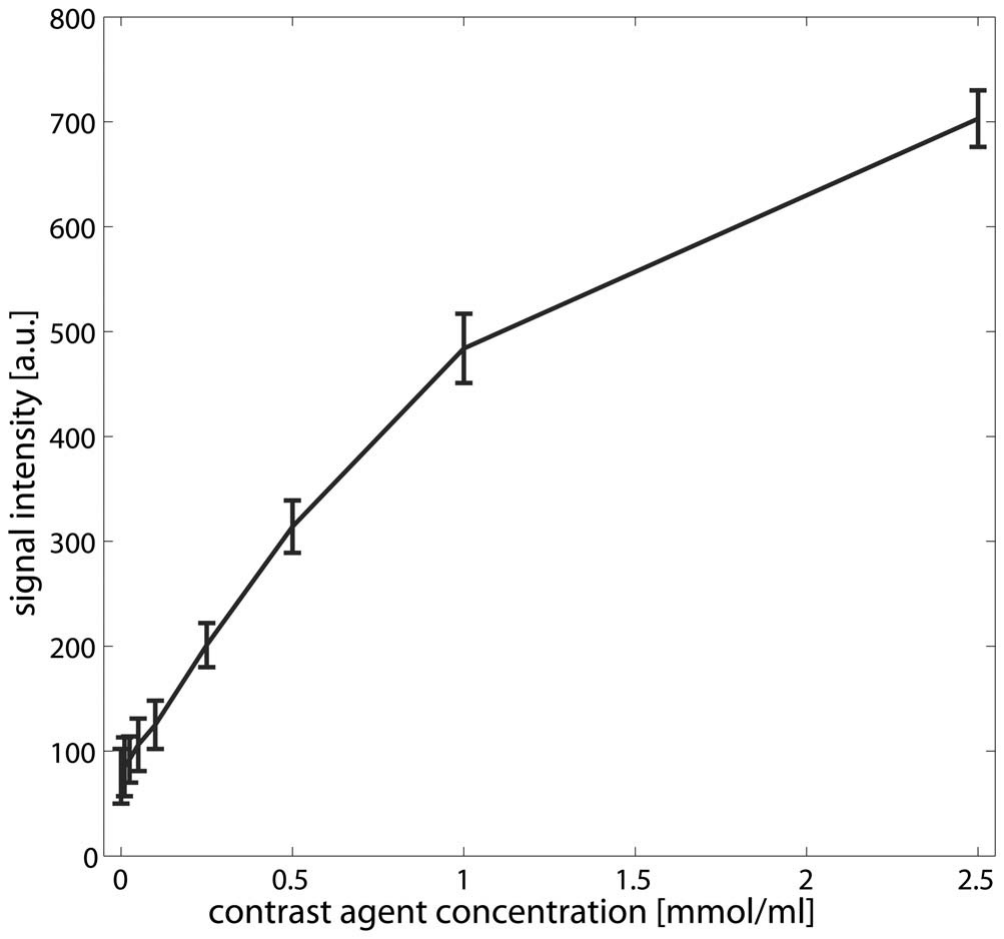
Plot of concentrations (mmol/ml) versus signal intensities (arbitrary units) obtained from a phantom consisting of tubes filled with water and different contrast agent concentrations (0, 0.01, 0.025, 0.05, 0.1 0.25, 0.5, 1.0, 2.5 mmol/ml). The error bars depict the standard deviation within a ROI of size 0.3^2^ placed within the tubes.

Figure 2 shows the plot of different contrast agent concentrations and respective signal intensities obtained by the DCE-MRI imaging protocol. Within the range between zero and 1 mmol/ml contrast agent a linear relationship between signal intensities and contrast agent concentration could be observed. Beyond 1 mmol/ml, a nonlinear relationship is observed.

**Figure 3:**
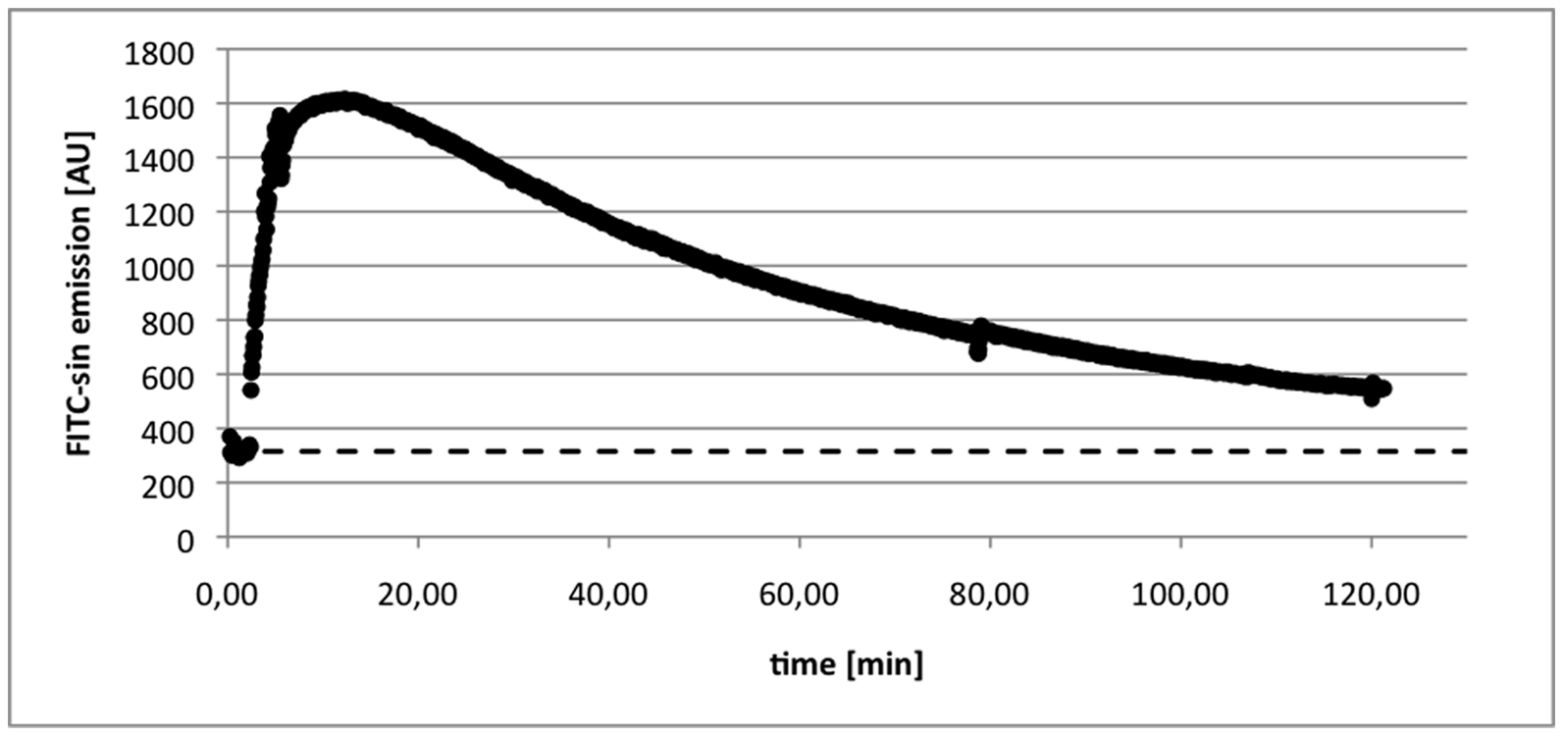
Example of FITC-sinistrin clearance curve from one animal. The dashed line depicts the background signal, which is subtracted from the signal for quantification of the GFR.

All animals were examined without problems; visual inspection of the MR images showed that signal cancellation caused by the optical device did not impair the post processing of the MR images. Figure 3 shows an example clearance curve obtained by the optical sensor.

**Figure 4:**
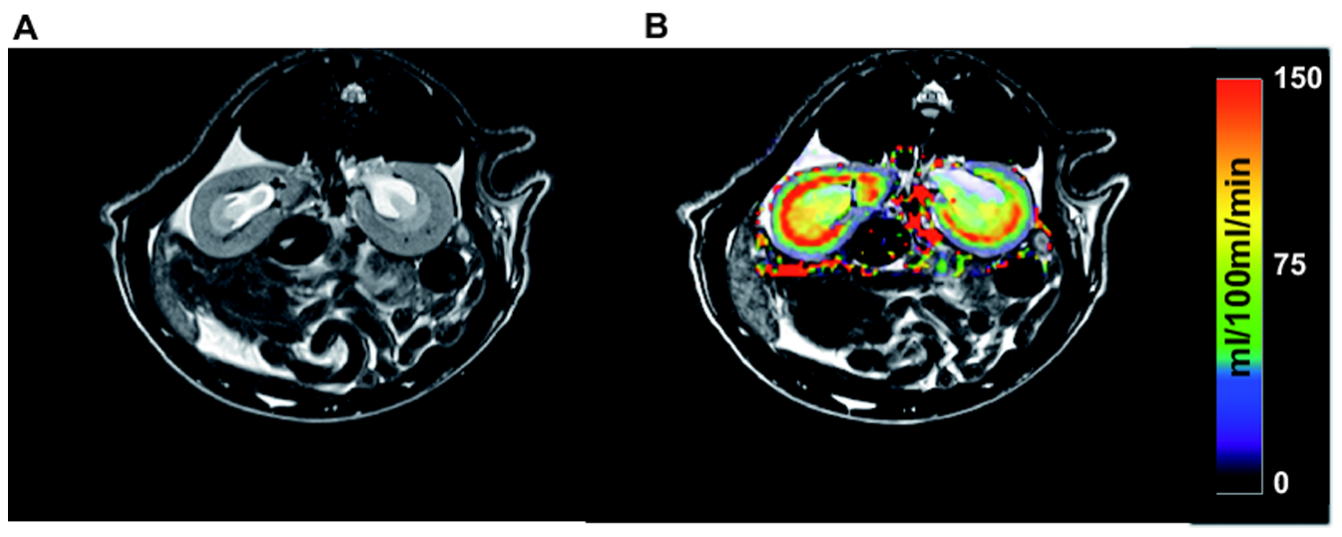
Image examples obtained by the MR measurements. A) depicts one slice of a T2 weighted morphological MR image used for cortex delineation, B) shows a parametric map of the tubular flow (filtration) calculated from the DCE- MRI perfusion data in units of ml/min/100 ml tissue. The map is superimposed onto the corresponding slice of the T2 weighted image.

**Figure 5:**
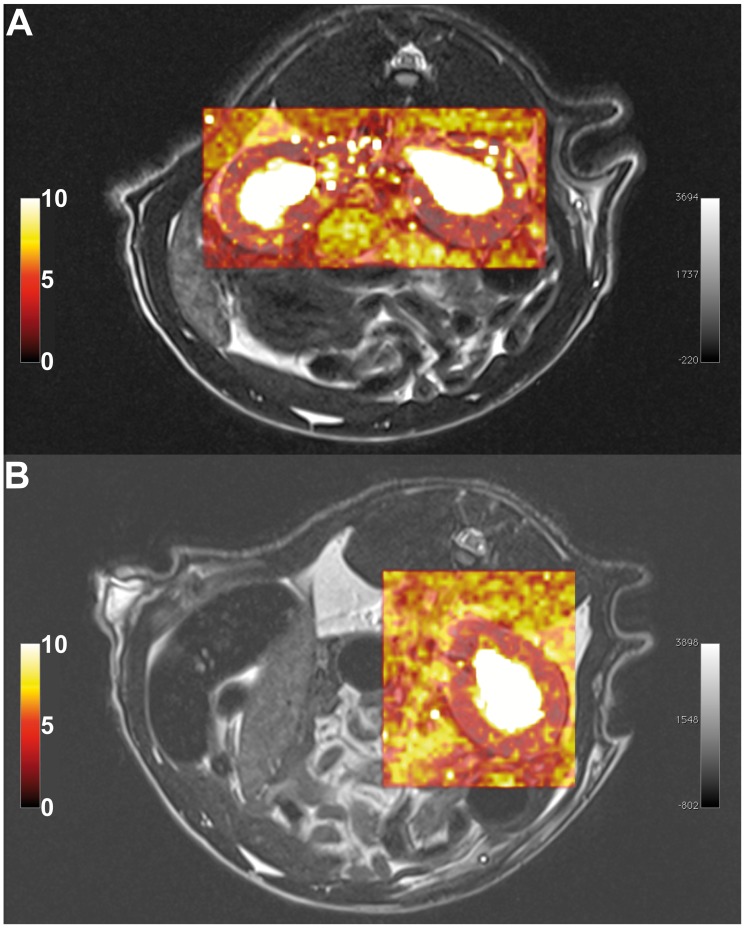
Example of colour-coded goodness of fit maps of the kidney for a) a healthy SD rat and b) a UNX rat. Fit error maps (χ^2^) are superimposed to the corresponding slice of the T2 weighted morphological image for better visualization.

**Figure 6:**
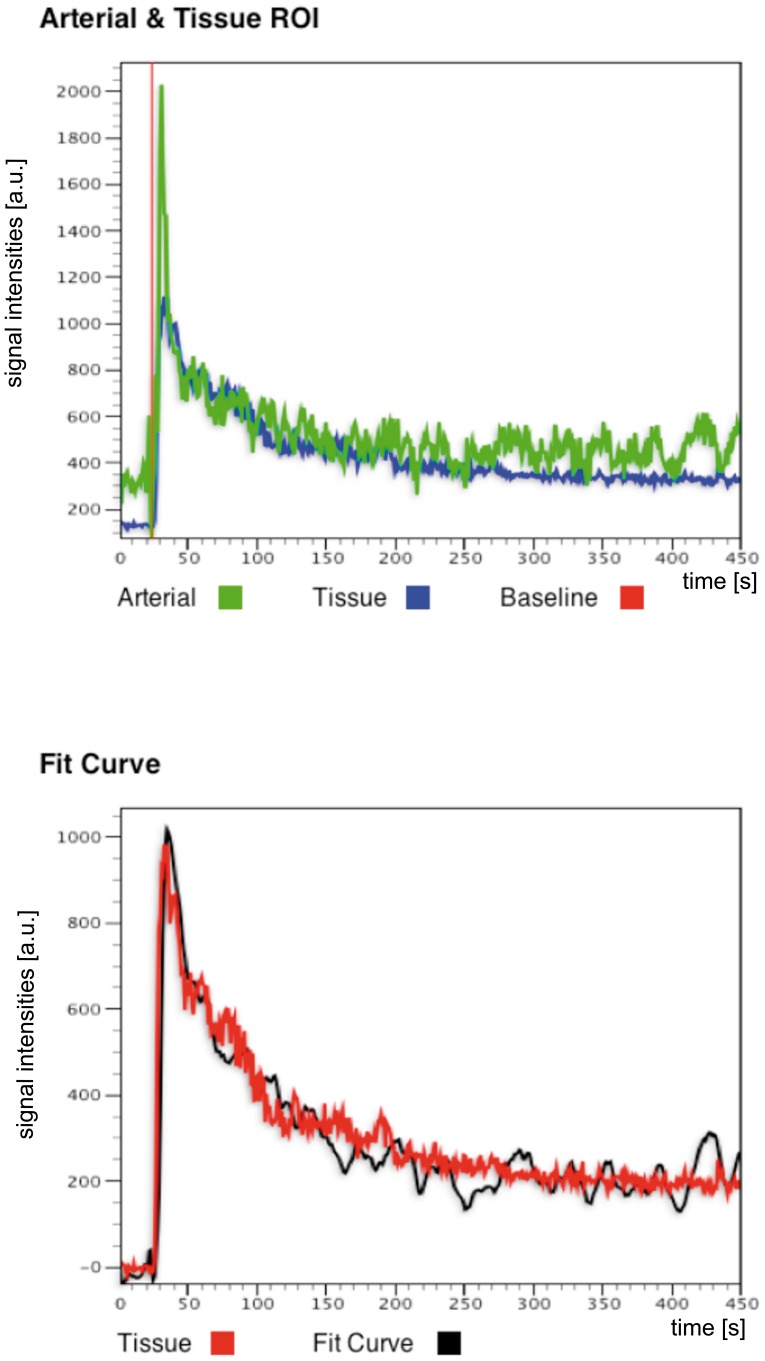
Example of fitting the 2CFM to a signal intensity curve. The data was derived from a ROI in the cortex of the left kidney of a healthy SD rat. Top row: Tissue signal intensity curve of cortex ROI (blue) and AIF (green). Red line depicts base line selection for signal normalization. Lower row: corresponding fit of the 2CFM (black) to the tissue signal intensity curve (red).

Figure 4 shows a T2 weighted high resolution MR image along a tubular flow parametric map derived from the 2CFM. Based on the T2 weighted image, the delineation of the cortex was performed and the ROIs were than transferred onto the parametric maps. Visual inspection of the χ^2^ maps (goodness of fit) automatically calculated by our software for each analysed data set show homogenous distribution of fit errors with only a few single voxels exceeding a high fit error, suggesting successful model fitting (see Fig. 5 for an example of a healthy SD rat and an UNX rat). The χ^2^ error averaged over each kidney cortex and data set is 7 ± 3. Similarly, Fig. 6 shows a signal intensity curve derived from a ROI in the renal cortex, a typical AIF derived from abdominal aorta, and the corresponding model fit of the 2CFM.

Table 1 summarizes the following parameters of healthy and UNX rats: kidney weight, kidney water displacement, mean cortical volume, total kidney volume and mean tubular flow as assessed by MRI.

**Table 1:** Kidney volumes for healthy and UNX rats estimated by weighting, water displacement and manual segmentation of MR images.

group	Kidney weight [g]		Water displacement [ml]		Cortical volume - MRI [ml]		Total volume – MRI [ml]		Mean tubular flow – MRI [ml/100 ml/min]	
	right	left	right	left	right	left	right	left	right	left
SD	1.36 (±0.13)	1.42 (±0.12)	1.40 (±0.12)	1.41 (±0.11)	1.42 (±0.25)	1.50 (±0.26)	1.95 (±0.34)	2.17 (±0.34)	68 (±37)	61 (±35)
UNX	1.76 (±0.38)		1.74 (±0.33)		1.63 (±0.45)		2.40 (±0.43)		73 (±18)	

Unilateral nephrectomy was performed on the left kidney. In case of the MRI, also cortical volumes were estimated for GFR calculations. Values are mean ± standard deviation.

Mean GFR for the transcutaneous measurement was 4.34 ± 2.22 ml/min (healthy SD rats) and 2.34 ± 0.90 ml/min (UNX rats) whereas GFR by DCE-MRI was 2.10 ± 0.64 ml/min (SD rats) and 1.17 ± 0.38 ml/min (UNX rats), respectively. The GFR values measured with both techniques for both groups are given in Table 2 and 3.

**Table 2:** Comparison of GFR values obtained by MRI and FITC-sinistrin techniques in healthy SD rats.

SD	GFR [ml/min]	
	transcutaneous FITC-sinistrin clearance	DCE-MRI
1	7.23	2.22
2	4.67	2.25
3	5.48	2.63
4	2.58	2.48
5	1.72	1.32
6	3.24	1.52
Mean (sd)	4.34 (± 2.22)	2.10 (± 0.64)

Mean GFR between methods differ by a factor of two.

**Table 3:** Comparison of GFR values obtained by MRI and FITC-sinistrin techniques in UNX SD rats.

UNX SD	GFR [ml/min]	
	transcutaneous FITC-sinistrin clearance	DCE-MRI
1	2.30	0.59
2	2.78	1.33
3	1.16	0.84
4	1.40	1.21
5	3.11	1.55
6	3.32	1.50
Mean (sd)	2.34 (± 0.9)	1.17 (± 0.38)

Mean GFR between methods differ by a factor of two.

**Figure 7:**
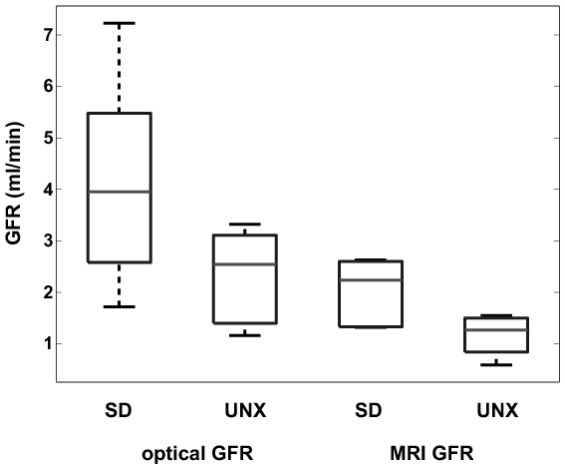
Boxplot of GFR values for each group and method. Differences between healthy and UNX rats are clearly visible as well as the offset between GFR measured by FITC-sinistrin and DCE-MRI. The boxes represent the 25th–75th percentiles, the horizontal lines within the boxes represent the median. The whiskers indicate 1.5 times the interquartile range (IQR) from the first quartile and 1.5 times the IQR from the third quartile; the circles represent outliers outside the whiskers.

**Figure 8:**
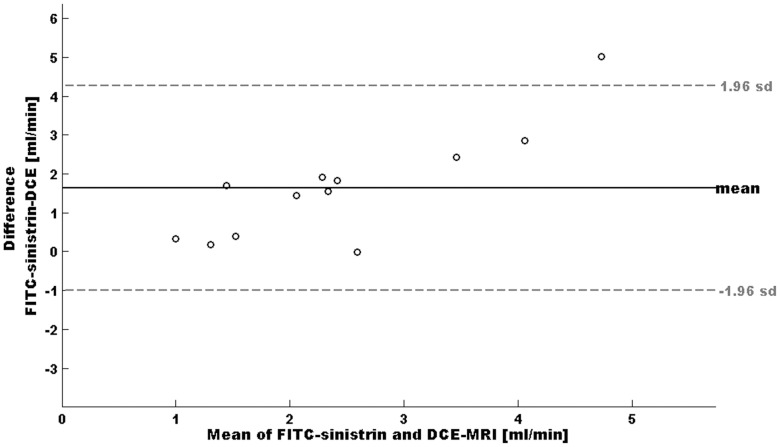
Bland-Altman plot of GFR values estimated by both methods. On the x-axis, the average of both methods is plotted whereas the y-axis shows the differences between the methods.

Using the transcutaneous method the UNX group shows a 46.1% lower GFR than the healthy SD group. Using the DCE-MRI technique the GFR of the UNX SD group shows a reduction in GFR of 44.3% compared to the healthy SD group. In both cases these differences are significant (healthy SD rats p = 0.037, UNX SD rats p = 0.011). However, there is a discrepancy in the overall GFR values by roughly a factor of two observed within the techniques in both groups. This is also reflected by the boxplots (Fig. 7) and the Bland-Altman plot (Fig. 8). For all data, the mean difference between methods is 1.6, the standard deviation 1.4. With exception of one rat, all data lie within the range of ±2 standard deviation.

## Discussion

In this study, we investigated the feasibility of simultaneously measuring GFR by two non-invasive techniques, namely DCE-MRI and transcutaneous FITC-sinistrin clearance. This approach enables the validation of MRI methods for GFR assessment in animals and, for the first time, a clearance procedure comparable to classical sinistrin [12], [13], [14] that is independent of blood/ urine sampling or laboratory assays.

Almost equal percentage differences in mean GFR between the healthy and the UNX SD rats were noted with both techniques (Table 2, 3 & Fig. 7). The discrimination between healthy animals and animals with reduced kidney function was significant for both techniques.

Furthermore, our results show a higher degree of agreement between methods than previously noted [16]. Mean bias was 1.6 and most data lie within ±2 standard deviations (see Fig. 8). Systematic errors were not detected. However, the mean GFR values of this study obtained with the transcutaneous FITC-sinistrin clearance are about twice as high as compared to the DCE-MRI values. The GFR values assessed with the optical imaging approach are in the expected range for anaesthetized rats when compared to results obtained from other groups and our prior studies. In these studies mean GFR values between 0.8 – 1.2 mL/100 g bw in 300 –450 g rats were found [20], [27], [28], [29], [30].

Nevertheless, in this work a significant improvement was achieved. At first a simultaneous measurement of GFR was achieved and the difference between methods was reduced to a factor of two. Furthermore, Bland-Altman plots showed no systematic errors. In addition both methods equally reflect the reduction in GFR when applied to the UNX rat model. In contrast, in the work of Sadick et al., GFR values differ by a factor of ten and the Bland-Altman plot show systematic errors [16].

One possible reason the DCE-MRI and FITC-sinistrin measurements were in better agreement in this study is that they were acquired simultaneously, whereas in the previous study by Sadick et al. the two measurements were acquired on consecutive days. Thus, a second anaesthesia administration was needed. It is known that anaesthesia influences the physiology of the animals causing changes also in the GFR [31], [32], [33]. This possible anaesthesia related bias was removed in the present study.

The accurate estimation of GFR by MRI requires the estimation of the cortical volume [26], [34]. In our study, DCE-MRI was performed by a 3D volume acquisition covering the whole kidney. In addition, high-resolution T2 weighted images, covering the same volume as the DCE-MRI acquisition were acquired to support the volumetry of the kidneys and renal cortex. Though no kidney volume measurement by histology (stereology) was performed, we also delineated the whole kidney volume (excluding the renal pelvis) as a second reference (see Tab. 1). The percentage of cortical volume to total volume was within the range of 73% – 76% as published in the literature [35], [36], [37].

Furthermore, we did not acquire the images in coronal but transversal slice orientation which might improve delineations by an easier discrimination of cortex and medulla. Eventually, the voxel resolution of the 2D DCE-MRI sequence of Sadick et al. was approximately double (1.0×1.0×2.5 mm^3^) in size compared to ours (0.6×0.6×1.2 mm^3^) and therefore more prone to partial volume effects.

A possible source of differences between GFR measurements is the required use of an arterial input function by MRI. The AIF selection is critical for reliable DCE-MRI analysis and an optimal method for its selection is currently unresolved [15]. Cutajar et al. showed that different sized AIFs could significantly alter the estimation of renal blood flow (RBF) and GFR, though GFR seemed to be less affected than RBF [38]. Due to the small size of the rats the correct manual assessment of the AIF in the abdominal aorta is much more difficult. Specifically, the diameter of the abdominal aorta is generally only several voxels in diameter. Moreover, the artery may be positioned close to the vein such that the selected voxels may overlap both vessels, thereby possibly inducing an error to the arterial input function [39]. Therefore, to minimize the bias of AIF selection to the results of the GFR calculation (see Fig. 6), attention was paid during the data analysis, to carefully outline the ROI for AIF analysis. Other solutions to overcome the influence of the AIF might be a double bolus injection [40], a population based AIF [41], or techniques without the need for an AIF, i.e. reference region model approaches [42].

Another possibility that may contribute to the discrepancy in GFR between the two methods may be an underestimation of the tubular flow by the DCE-MRI as no conversion of signal intensities to concentration curves was performed; instead a linear relationship was assumed. This linear relationship was confirmed by phantom measurements with different concentrations of Dotarem (see Fig. 2) and a similar linear relationship was reported in a study of contrast agent clearance in rats by Bauman and Rudin [43]. This may influence the estimation of MRI-GFR [15] and in future research this influence should be analyzed. Nevertheless, in future research the influence of conversion of signals into concentrations should be analyzed.

In addition, there was no correction for movement artifacts due to the breathing of the animals. Due to such movement artefacts, the time intensity curve becomes noisy and may lead to incorrectly fitted data. Calculated fit error maps as well as fit curves (see Fig. 5 and 6) however, suggest correct fits. Nevertheless, incorporation of a movement correction as well as segmentation techniques might lead to even better results [44], [45] and therefore, are currently investigated by our group.

The higher overall values of GFR assessed with the transcutaneous technique compared to the DCE-MRI method may partly be explained by the underlying one compartment model, which is known to overestimate GFR to some extent [46]. Comparing the standard deviation of GFR within the groups of animals, the transcutaneous method showed a higher interanimal variation than DCE-MRI. However, previous studies by our group showed a much smaller standard deviation [20], [27]. This difference can be explained by the fact, that the anesthesia protocol compared to previous studies was changed to allow for better animal handling during MRI. However, it seemed to have a different influence on the single animal’s physiology and thus, the GFR and kidney function [31], [32].

In summary, the simultaneous acquisition of transcutaneous optical imaging and MRI may have a huge impact on the validation of existing and newly developed MRI methods for GFR assessment in animal models.
